# Nrf2 positively regulates autophagy antioxidant response in human bronchial epithelial cells exposed to diesel exhaust particles

**DOI:** 10.1038/s41598-020-59930-3

**Published:** 2020-02-28

**Authors:** Daniela Perroni Frias, Raquel Labiapari Nunes Gomes, Kelly Yoshizaki, Regiani Carvalho-Oliveira, Monique Matsuda, Mara de Souza Junqueira, Walcy Rosolia Teodoro, Pérola de Castro Vasconcellos, Daniela Cristina de Almeida Pereira, Paulo Roberto da Conceição, Paulo Hilário Nascimento Saldiva, Thais Mauad, Mariangela Macchione

**Affiliations:** 1 Laboratory of Experimental Air Pollution (LIM-05), Department of Pathology, School of Medicine, University of São Paulo, Av. Dr. Arnaldo 455, São Paulo, Brazil; 2 Laboratory of Investigation in Ophthalmology (LIM-33), Division of Ophthalmology, School of Medicine, University of São Paulo, Av. Dr. Arnaldo 455, São Paulo, Brazil; 3 Center for Translational Research in Oncology (LIM24), Cancer Institute of the State of São Paulo, Hospital das Clínicas (HCFMUSP) School of Medicine, University of São Paulo, Av. Dr. Arnaldo, 251, São Paulo, SP, Brazil; 4 Rheumatology Division of the Hospital das Clinicas da Faculdade de Medicina da Universidade de Sao Paulo FMUSP, São Paulo, SP, BR, Av. Dr. Arnaldo, 455, room 3124, Cerqueira César, São Paulo, SP, 01246-903 Brazil; 5 Chemistry Institute, University of São Paulo, São Paulo, Brazil; 6 Physicist. Researcher of clean energy alternatives and clean mobility. Inventor and freelancer of Laboratory of Experimental Air Pollution in University of São Paulo, São Paulo, Brazil

**Keywords:** Macroautophagy, Stress signalling

## Abstract

Diesel exhaust particles (DEP) are known to generate reactive oxygen species in the respiratory system, triggering cells to activate antioxidant defence mechanisms, such as Keap1-Nrf2 signalling and autophagy. The aim of this study was to investigate the relationship between the Keap1-Nrf2 signalling and autophagy pathways after DEP exposure. BEAS-2B cells were transfected with silencing RNA (siRNA) specific to Nrf2 and exposed to DEP. The relative levels of mRNA for Nrf2, NQO1, HO-1, LC3B, p62 and Atg5 were determined using RT-PCR, while the levels of LCB3, Nrf2, and p62 protein were determined using Western blotting. The autophagy inhibitor bafilomycin caused a significant decrease in the production of Nrf2, HO-1 and NQO1 compared to DEPs treatment, whereas the Nrf2 activator sulforaphane increased the LC3B (p = 0.020) levels. BEAS-2B cells exposed to DEP at a concentration of 50 μg/mL for 2 h showed a significant increase in the expression of LC3B (p = 0.001), p62 (p = 0.008), Nrf2 (p = 0.003), HO-1 (p = 0.001) and NQO1 (p = 0.015) genes compared to control. In siRNA-transfected cells, the LC3B (p < 0.001), p62 (p = 0.001) and Atg5 (p = 0.024) mRNA levels and the p62 and LC3II protein levels were decreased, indicating that Nrf2 modulated the expression of autophagy markers (R < 1). These results imply that, in bronchial cells exposed to DEP, the Nrf2 system positively regulates autophagy to maintain cellular homeostasis.

## Introduction

Chronic exposure to air pollution has been associated with adverse effects on the health of individuals^1^, such as inflammation^2–4^ and mucociliary clearance dysfunction^5^ of the respiratory system. Air pollution particulate matter (PM) induces oxidative stress in airway tissues, causing living organisms to activate their defences to prevent cell death^6–8^. One mechanism that cells use for defence against oxidative stress is autophagy, which is a homeostatic process that reduces cytoplasmic volume by degrading damaged organelles and proteins through a lysosome-dependent degradation process, and new organelles and proteins are synthesized as replacements^9–11^. Previous studies have emphasized that exposure to PM induces the generation of reactive oxygen species (ROS) and increases the levels of autophagy and cell death^12–14^.

Some of the most harmful components of urban PM are derived from diesel exhaust particles (DEP)^15,16^. Usually, composed of carbon nuclei, DEP have large surface areas that can adsorb other chemicals in the environment, such as polycyclic aromatic hydrocarbons (PAHs), sulphate, nitrate, metals, carbon monoxide, aldehydes and various low molecular weight hydrocarbons^17,18^. PAHs and their derivatives are especially important because they are able to generate ROS in tissues^19^.

Genes encoding key antioxidant enzymes are important for maintaining intracellular redox homeostasis through the antioxidant response element (ARE) found within the promoter regions of these genes^20^. The transcription factor nuclear factor erythroid-derived 2-like 2 (NFE2L2, also known as Nrf2) induces ARE genes as part of a protective response against oxidative challenge to organelles and macromolecules^21^.

Recent *in vivo* and *in vitro* models have shown that the oxidative damage caused by PM exposure can activate the Nrf2-antioxidant response element signalling pathway^22,23^. Additionally, A549 cell and murine alveolar macrophage cultures exposed to DEP have shown increased Nrf2 and HO-1 (an Nrf2 target gene) expression levels^24^.

There is evidence that some autophagy genes, such as Atg5, Atg4D and SQSTM1/p62, have ARE promoter regions, and these promotors may be regulated through Nrf2 activity^25^. Nrf2 is normally present in the cytoplasm attached to Kelch-like ECH-associated protein 1 (Keap1), which facilitates the ubiquitination and proteolysis of Nrf2. In addition, protein p62 interacts with the same binding site as that of Nrf2-Keap1 and competitively inhibits the Keap1-Nrf2 interaction. The accumulation of p62 within the cells allows it to interact with Keap1 more frequently, resulting in the inhibition of Keap1 and, consequently, the activation of Nrf2^26,27^. Also, p62 has been found to be a mediator in the formation of protein aggregates for autophagy recycling, functioning as an adapter for facilitating the binding of ubiquitinated protein aggregates and delivering them to autophagosomes, by associating with LC3 (protein light chain 3) a protein in autophagosome membrane^28^

The aim of this study was to examine the role of DEP in the Nrf2/ARE-mediated oxidant response and the influence of this pathway on the induction of autophagy in the human BEAS-2B bronchial cell line.

## Results

### DEP characterization

To verify the composition of the DEP collected, two characterization assays were performed: EDX (X-ray fluorescence by the energy dispersive method) to determine elements that comprise the DEP (Table 1); high-performance liquid chromatography to separate the PAH fractions and their derivatives; and gas chromatography in conjunction with mass spectrometry (GC/MS) was chosen for identification and quantification (Table 2). The results show high concentrations of PAH and metals in the DEP sample.

**Table 1:** Elementary compounds of DEP.

Element	EDX	Element	EDX
Cr	16,14 ± 11,20	Ba	24,78 ± 15,68
Mn	22,62 ± 1,34	Pb	2,29 ± 1,60
Fe(%)	0,29 ± 0,00	Na(%)	0,21 ± 0,03
Co	79,85 ± 5,47	Mg	3,99 ± 6,06
Ni	3,27 ± 1,73	Al	0,67 ± 0,58
Cu	14,09 ± 1,15	Si	338,24 ± 17,07
Zn	285,21 ± 0,51	P	318,49 ± 3,89
As	2,94 ± 1,59	S(%)	0,27 ± 0,00
Br	2,05 ± 1,30	K	73,29 ± 2,51
Rb	1,35 ± 0,59	Ca(%)	0,18 ± 0,00
Zr	1,43 ± 0,38	Sc	150,03 ± 3,97
Cd	1,01 ± 0,01	Cl	5,20 ± 1,36

The quantity of elements is calculated in ppm (part per million), except those indicated by “%”. The values presented are mean ± SEM (standard error mean).

**Table 2:** Result of organic fraction of DEP by gas chromatography.

HPA	Recovery (%)	Result (ng/g)	
Fluorene	74	11	
Phenanthrene	96	47	
Anthracene	53	7	
Fluoranthene	81	35	
Pyrene	74	32	
Benzo(a)anthracene	65	8	
Criseno	66	15	
BbF	73	32	
BkF	70	11	
BeP	74	25	
BaP	69	22	
InP	85	6	
DBA	93	13	
BPer	88	53	
Cor	92	170	
**Elemental Carbon (EC)**	**Organic Carbon (OC)**		
69,30%	30,7		
	Oca	OCb	PC
	0,3	3,9	26,6

The acronyms correspond respectively to the compounds: Benzo (b) fluoranthene (BbF), Benzo (k) fluoranthene (BkF), Benzo (e) pyrene (BeP), Benzo, 3-cd) pyrene (Ind), Dibenzo (a, h) anthracene (DBA) and Benzo (ghi) perylene (BPe).

### DEP cause disruptions to the respiratory chain and reduce cell viability

**Figure 1:**
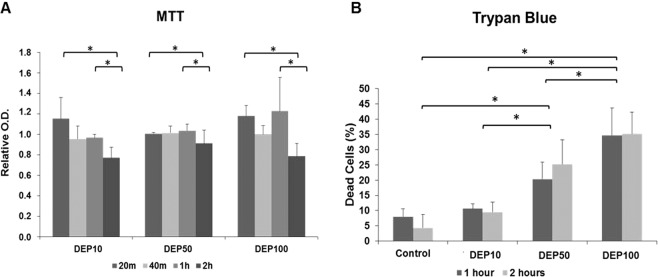
Cytotoxicity Assays: (**A**) Results from the analysis of formazan absorbance produced by cells exposed only to LHC-9 culture medium (control), DEP at 10 μg/mL, DEP at 50 μg/mL, and DEP at 100 μg/mL. A significant difference was found for exposure times (p = 0.015). Results from the Holm-Sidak test showed a difference between the times of 20 min and 2 h (p = 0.009) and between the times of 1 h and 2 h (p = 0.01) for the groups exposed to the DEP. (**B**) Results from the analysis of the percentage of dead cells that had been exposed only to LHC-9 culture medium (control), 10 μg/mL DEP or 50 μg/mL DEP. A significant difference was found for concentrations (p = 0.004). The Holm-Sidak method showed a difference between groups: 100 μg/mL vs. the control (p = 0.009); 100 μg/mL vs. 10 μg/mL (p = 0.010); 100 μg/mL vs. 50 μg/ml (p = 0.025); 50 μg/mL vs. the control (p = 0.007); and 50 μg/mL vs. 10 μg/ml (p = 0.015).

To ensure that the concentration of the DEP to be used in subsequent experiments did not cause a high death rate among the BEAS-2B cells, we performed MTT and Trypan blue tests. The MTT test is a measure of metabolic activity, while the Trypan blue test is a direct measure of the cell death rate. The results of the MTT assay showed that DEP exposure significantly reduced formazan absorbance, with significant differences between the 20 min and 2 h groups (p = 0.009) and between the 1 and 2 h groups (p = 0.01) (Fig. 1A). The results of the Trypan blue assay indicate that exposure to 50 µg/mL DEP significantly increased the percentage of dead cells compared to the number in the control (p = 0.007) and 10 µg/mL (p = 0.015) groups. Additionally, exposure to 100 µg/mL DEP concentration for 2 h increased the percentage of dead cells among all groups: 100 µg/mL vs. the control (p = 0.009); 100 µg/mL vs. 10 µg/mL (p = 0.010); 100 µg/mL vs. 50 µg/mL (p = 0.025) (Fig. 1B). Taken together, these results allowed the decision for the concentrations of 10 µg/mL and 50 µg/mL, which did not show a greater than 30% reduction in cell viability.

### DEP increase the mRNA expression of both autophagic and antioxidant genes

The cells were exposed to DEPs at 10 and 50 µg/mL concentrations (DEP10 and DEP50) for 2 h. Along with DEPs exposure, an RT-PCR assay was performed to verify the autophagy and antioxidation markers using autophagy and Nrf2 system controls. The following substances were used as controls: 1) EBSS to induce starvation in cells; 2) sulforaphane at a 10 µM concentration for 2 h to activate the Nrf2 system; and 3) bafilomycin at a 10 nM concentration for 4 h to inhibit autophagic flux. We analysed the following genes: 1) LC3B, p62 and Atg5 in the autophagy pathway and 2) Nrf2, NQO1 and HO-1 in the antioxidant pathway.

**Figure 2:**
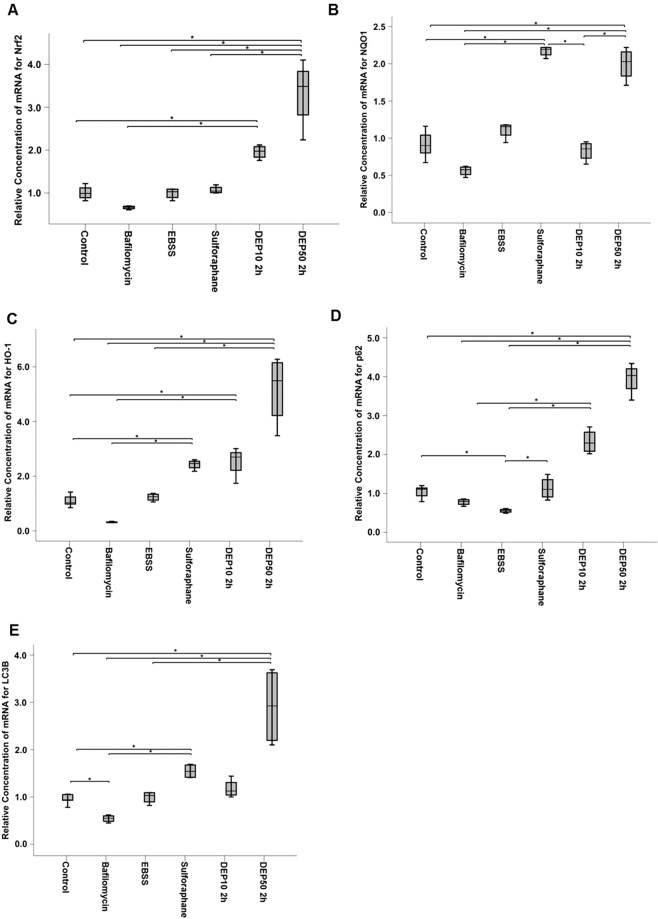
Results of gene expression analysis (RT-PCR) for the autophagy and Nrf2-system markers after exposure to 10 and 50 μg/mL DEP for 2 h, EBSS for 40 min, 10 nM bafilomycin for 4 h and 10 μM sulforaphane for 24 h. The quantities were calculated by the 2^−ΔΔCT^ method. (**A**) Nrf2: there was a significant difference (p < 0.001), and the pairwise test showed a difference between treatments: DEP50 vs control, (p = 0.003), bafilomycin (p < 0.001), EBSS (p = 0.011) and sulforaphane (p = 0.042); DEP10 vs control (p = 0.03) and bafilomycin (p < 0.001). (**B**) NQO1: there was a significant difference (p < 0.001), and the pairwise test showed a difference between DEP50 vs control (p = 0.015), bafilomycin (p < 0.001) and DEP10 (p = 0.017); sulforaphane vs control (p = 0.005), bafilomycin (p < 0.001) and DEP10 (p = 0.006); bafilomycin vs EBSS (p = 0.010). (**C**) HO-1: there was a significant difference (p < 0.001), and the pairwise test showed a difference between DEP50 vs control (p = 0.001), bafilomycin (p < 0.001) and EBSS (p = 0.017); DEP10 vs control (p = 0.015) and bafilomycin (p = 0.001); sulforaphane vs control (p = 0.41) and bafilomycin (p = 0.003); (**D**) p62: there was a significant difference (p < 0.001), and the pairwise test showed a difference between: DEP50 vs control (p = 0.008), bafilomycin (p = 0.001) and EBSS (p < 0.001); DEP10 vs bafilomycin (p = 0.009) and EBSS (p < 0.001); EBSS vs control (p = 0.026) and sulforaphane (p = 0.022). (**E**) LC3B: there was a significant difference (p < 0.001), and the pairwise test showed a difference between DEP50 vs control (p = 0.001), bafilomycin (p < 0.001) and EBSS (p = 0.18); bafilomycin vs DEP10 (p = 0.13) and sulforaphane (p = 0.001); control vs sulforaphane (p = 0.020).

The results showed that DEP50 treatment lead to increased gene expression for both Nrf2-system and autophagy markers (Fig. 2A–E), mainly when compared with autophagy controls EBSS and bafilomycin. DEP10 treatment increased Nrf2, HO-1 and p62 levels, mainly when compared with control and bafilomycin treatments (Fig. 2A,C,D), indicating that both treatments are able to interfere in both pathways. Interestingly, Nrf2-system induction by sulforaphane increased the expression of the autophagy markers p62 and LC3B (Fig. 2D,E), mainly when compared to control, whereas autophagy impairment by bafilomycin negatively interfered with the expression of Nrf2-regulated proteins: Nrf2, NQO1 and HO-1 (Fig. 2A–C), mainly when compared to DEP50 treatment. These results indicate that were able to interfere in both pathways (Fig. 2A–E).

### Nrf2 positively regulates autophagy markers in DEP exposure

**Figure 3:**
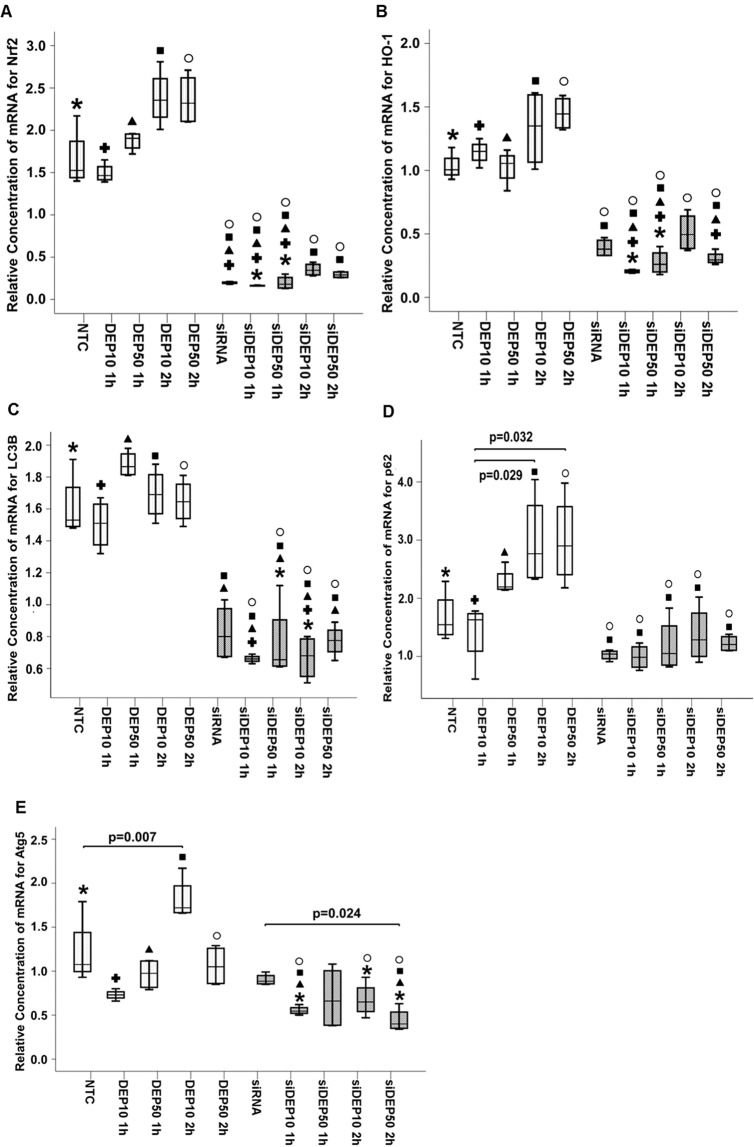
Expression of autophagy and antioxidant markers in Nrf2-specific siRNA-transfected BEAS-2B cells exposed to DEP at concentrations of 10 μg/mL and 50 μg/mL for 1 h and 2 h. The relative concentration of mRNA markers was determined by the 2^−ΔΔCT^ method. The results of the Kruskal-Wallis test showed a significant difference between the cells transfected with siRNA and non-transfected cells for the following genes: (**A**) Nrf2 (p < 0.001); (**B**) HO-1 (p < 0.001), (**C**) LC3B (p < 0.001), (**D**) p62 (p = 0.001), and (**E**) Atg5 (p = 0.001). The pairwise multiple comparison test showed the differences between paired groups. The symbols “*”, “+”, “▲”, “■” and “○” represent the NTC, DEP10 1 h, DEP50 1 h, DEP10 2 h, and DEP50 2 h groups, respectively. Above the siRNA and siDEP group box plots, the list of symbols indicates significant results compared to the results of the NTC and DEP groups. The values of p for the pairs are described in Tables 5–9 in the online resource.

Silencing RNA (siRNA) specific to Nrf2 was used to reduce its activity in the BEAS-2B cells. After 48 h of culture for transfection, the cells were exposed to DEP at concentrations of 10 and 50 µg/mL for 1 h and 2 h. The results showed that siRNA treatment significantly reduced the amount of p62 (p = 0.001) (Fig. 3D) and LC3B (p < 0.001) (Fig. 3C). The non-transfected BEAS-2B cells exposed to DEP at a concentration of 10 μg/mL for 2 h had increased Atg5 mRNA levels (p = 0.007) (Fig. 3E), indicating that DEP induced Atg5 when Nrf2 was active in the cells. However, when Nrf2 was reduced through siRNA, there was a significant Atg5 decrease in the BEAS-2B cells transfected with siRNA compared with that in the BEAS-2B cells exposed to siDEP50 for 2 h (p = 0.024), thereby confirming that the Atg5 response is dependent on Nrf2.

### siRNA treatment reduced the protein amount

**Figure 4:**
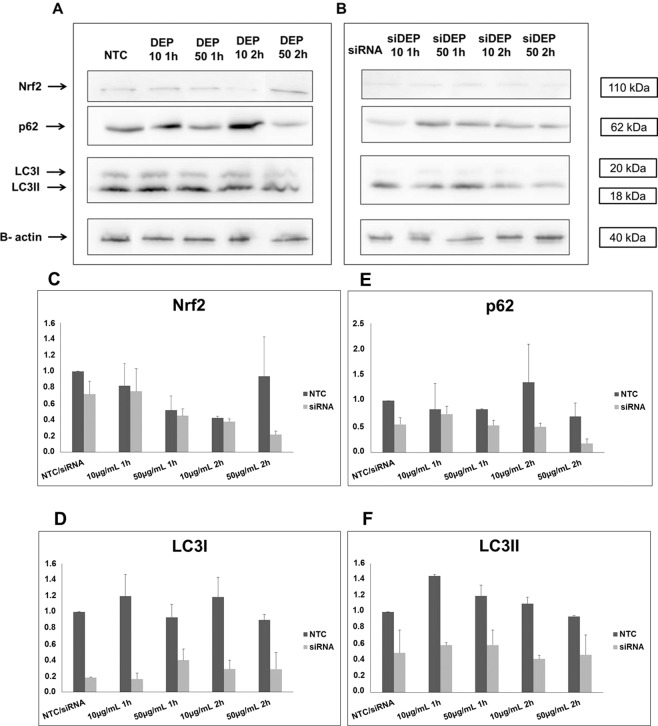
Proteins in the PVDF membranes as revealed by chemiluminescence. Samples were derived from the same experiment, and both gels were processed in parallel. The autophagy markers p62 and LC3I and II were analysed along with the expression of Nrf2. (**A**) Groups of cells transfected with the negative control and exposed to DEP at concentrations of 10 and 50 μg/mL for 1 h and 2 h. All proteins in these groups were in the same membrane. (**B**) Groups of cells transfected with siRNA for Nrf2 and exposed to DEP at concentrations of 10 and 50 μg/mL for 1 h and 2 h. All proteins shown are in the same membrane. The ratio of protein expression was determined by normalizing the expression of each protein of interest to that of β-actin and the normalized value ratio to that of the control value (NTC). Thus, R (ratio) >1 is considered to indicate increased protein level and R < 1 indicates decreased protein level. All R values are shown in tabular form in the online resources (Table 10).

To determine the proportion of each protein expressed under these conditions, Western blotting was performed with Nrf2, LC3B and p62 (Fig. 4). When compared to the levels of the BEAS-2B NTC cell groups, an evident decrease in these proteins was observed for all the siRNA-transfected BEAS-2B cell groups. For the BEAS-2B NTC cells, the results from the analysis of the Western blot bands showed an increase in Nrf2 after 2 h of exposure to DEP50 (R = 1.43) (Fig. 4C) and an increase in p62 in the BEAS-2B cells exposed to DEP10 (R = 1.3 for 1 h and R = 2.1 for 2 h) (Fig. 4E). The amount of LC3II — the functional form of LC3B — was increased in the DEP10 1 h group (R = 1.46) and DEP50 1 h group (R = 1.3) (Fig. 4F). LC3II was decreased in the siRNA-transfected BEAS-2B cells exposed to DEP10 for 1 h, DEP10 for 2 h and DEP50 for 2 h to a greater extent than it was in the cells treated with siRNA alone (Fig. 4F).

### Autophagosomes were reduced in the siRNA-transfected cells

**Figure 5:**
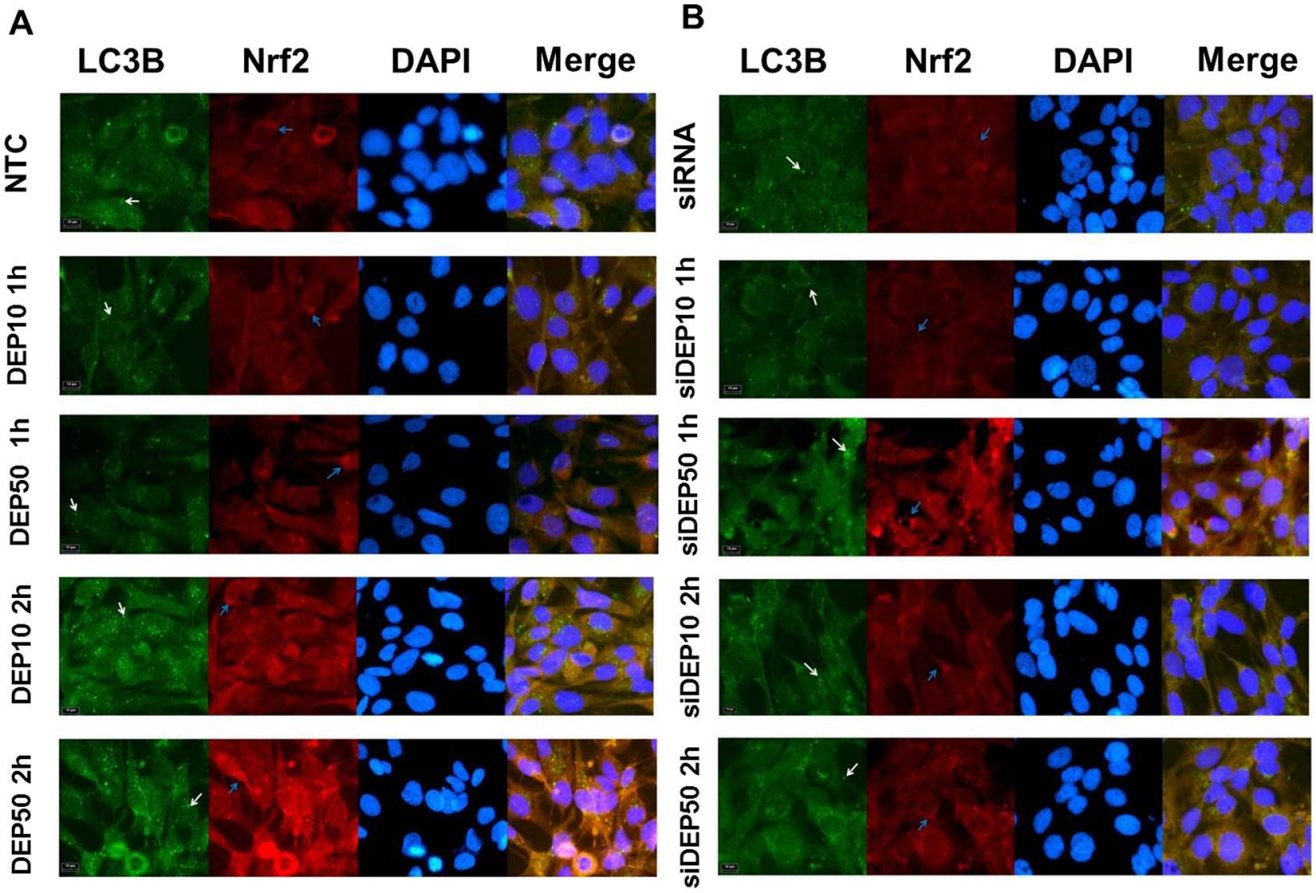
Results of the immunofluorescence method: (**A**) cells transfected with the negative control and (**B**) cells transfected with siRNA groups. The blank arrows in the LC3B and Merge columns indicate more intense green spots corresponding to the autophagosomes. The blue arrows in the Nrf2 column indicate some positive markers, for which the red fluorescence is more intense. In the last column, Merge indicates the overlap of the green (LC3B), red (Nrf2) and DAPI images. The yellowish colour in the Merge image indicates the overlapping of the red and green colours, whereas the more intense green spots correspond to autophagosomes. Images were acquired with an Olympus BX51 microscope at 40× and 2× magnification using Image Pro Plus software. Merged images were generated using ImageJ software.

An immunofluorescence assay was performed with the BEAS-2B cells to verify autophagosome formation and Nrf2 protein expression (Fig. 5). The yellow appearance after the images were merged (Merge) indicates the overlap between the green and red markers; i.e., both proteins were present in the cell: LC3B (green) and Nrf2 (red). As shown in Fig. 5A, autophagosomes are identified as bright green dots in both the LC3B and the Merge columns. The DEP10 2 h and DEP50 2 h groups had a higher number of green spots compared to that in the other groups, indicating that the autophagy was more intense in these groups. In the merged image of the DEP50 2 h group, the orange tone indicates the high intensity of the red spots (Nrf2). Figure 5B shows the BEAS-2B cells transfected with siRNA. The red staining is less intense because of Nrf2 suppression. LC3B is represented by positive green dots, and although less intense, they indicate that autophagy remained active when the cells were transfected with siRNA; however, autophagy remained much less active compared that in the non-transfected BEAS-2B cells.

## Discussion

In the present study, BEAS-2B cells were exposed to DEP at different times and concentrations to assess autophagy and antioxidant responses. The results indicate that DEP decrease cell viability and elicit the response of both biochemical pathways. The results of this study showed that the DEP concentrations of 10 and 50 μg/mL for 2 h of exposure can activate both the antioxidant pathway — increasing the expression of Nrf2, HO-1 and NQO1 — and the autophagy pathway — increasing p62 and LC3B expression. However, there were different responses between the two concentrations, which may be explained by particles behaviour in liquid solutions, since according to Seriani *et al*.^29^ at higher concentrations DEP tends to form large clusters while lower concentrations can penetrate epithelial cells more easily and fast, which may cause a different reaction in epithelium. In addition, siRNA treatment showed that autophagy is downregulated when the Nrf2 transcription factor showed low activity, indicating a positive relationship between the two mechanisms in maintaining cell survival in BEAS-2B cells exposed to DEP.

Nrf2 is a transcription factor that regulates a series of proteins with promoter regions that have an ARE motif, including some genes related to autophagy, such as p62 and Atg5^25^. The results of the present study indicate that Nrf2 activation positively regulates DEP-induced autophagy at the transcriptional level, as indicated by the considerable decrease in autophagy markers when Nrf2 was inhibited by siRNA, even during DEP exposure. Nrf2 suppression caused a significant decrease in the relative amounts of p62 and LC3B mRNA and decreased the relative protein levels of p62 and LC3II (Fig. 4). On the other hand, in the controls, sulforaphane-induced Nrf2 caused increased expression of Atg5, LC3B and p62. Taken together, the results showing the response of these autophagy markers — decreasing when Nrf2 is inhibited or increasing when Nrf2 is expressed — suggest that this transcription factor positively controls the transcription of these genes.

The immunofluorescence analysis results showed that the siRNA treatment reduced the frequency of green spots, as observed when the groups shown in Fig. 5A were compared to those shown in Fig. 5B. The BEAS-2B cells exposed to DEP for 2 h had yellow spots in Merge [a result of the overlapping LC3B (green) with Nrf2 (red)], greater intensity of the red dots representing Nrf2 and the green spots representing LC3B, attesting to the fact that DEP can induce both autophagy and the antioxidant pathway at the same time. The siRNA-transfected BEAS-2B cell groups showed a less intense yellow in the merged image than did the BEAS-2B cells not transfected with siRNA. The autophagy markers that were observed in the siRNA-transfected BEAS-2B cell groups can be considered long-lasting proteins that had already been produced in the cell prior to the siRNA transfection and may have been recycled by the autophagy mechanism itself, as indicated by the low levels of LC3B, Atg5 and p62 mRNA, which do not reflect *de novo* synthesis of these proteins.

Autophagy induction has been linked to air pollution exposure. Lai *et al*.^30^ exposed A549 cells to DEP, urban dust and black carbon and found a significant increase in autophagy as indicated by the conversion of LC3I to LC3II. Long *et al*.^14^ demonstrated that exposure of bronchial cells to 2.5 µm PM induced autophagy by overexpressing Atg5 and beclin-1, increasing the ratio of LC3I to LC3II proteins and increasing autophagosome formation. Bai *et al*.^12^ showed that PM_1_ (1 μm) at a concentration of 100 μg/mL induces autophagy and intracellular oxidative stress in type II alveolar epithelial cells in a dose-dependent manner. The authors also pre-treated the cells with antioxidants and chelators to show that the oxidative stress promoted by the particles was generated by the metals present in the PM composition (Fe, Cu and Mn). These pre-treatments also reduced the induction of autophagy in these cells compared to that in the cells treated with PM_1_ only. However, few studies have reported the participation of Nrf2 in the regulation of autophagy after PM or DEP exposure.

The relationship between the autophagy and antioxidant pathways mediated by Keap1-Nrf2 has been addressed in several studies using different substances. Wang *et al*.^31^ used cadmium-induced malignant transformation of BEAS-2B cells and found deficient autophagy levels, accumulation of p62 and constitutive activation of Nrf2 in these cells. However, sulforaphane treatment was able to restore autophagic flux and decrease Nrf2 expression in the transformed cells. In normal BEAS-2B cells, sulforaphane promoted Nrf2 activation, increased autophagy levels and decreased ROS levels, indicating that sulforaphane (and, as a consequence, Nrf2 activation) plays a dual role in regulating autophagy.

Son *et al*.^32^ used nickel-transformed BEAS-2B cells and showed increased LC3II levels and overexpression of Stat3, a protein with a promoter region that has an ARE binding site. When Stat3 levels were suppressed, a reduction in LC3II level was found, while Nrf2 suppression resulted in depletion of Stat3, indicating that Nrf2 exerts an influence on the sensitivity of autophagy. Shah *et al*.^33^ demonstrated that arsenic induces p62 expression *in vivo* and *in vitro*. Keratinocytes were transfected with siRNA specific to Nrf2, resulting in the suppression of p62 expression, even when the keratinocytes were exposed to arsenic. The authors concluded that Nrf2 is the transcription factor critical for p62 expression. To elucidate the positive relationship between p62 and Nrf2, the authors treated the cells with siRNA for p62, having found decreased expression of HO-1, NQO1 and GCLC proteins, which each had a promoter region activated by Nrf2, indicating that Nrf2 depends on p62 to be activated and vice versa.

However, in some studies, Nrf2 was associated with an inhibitor of autophagy. Lau *et al*.^34^ demonstrated that arsenic exposure in BEAS-2B cells activated Nrf2, disrupting autophagic flux and the accumulation of p62 and LC3B, resulting in a connection between Keap1 and p62. Additionally, when cells were treated with sulforaphane, a Nrf2 activation promoter, the autophagosome levels decreased. Zhu *et al*.^35^ demonstrated that exposure of BEAS-2B cells to cigarette smoke extract (CSE) increased the expression of LC3I and LC3II, p62 and NQO1. However, increased Nrf2 expression via inhibition of Keap1 resulted in decreased expression of LC3B, whereas siRNA specific to Nrf2 restored the levels of LC3I and II in the cells exposed to CSE, suggesting that Nrf2 activation can negatively regulate autophagy.

The divergence found in these studies indicates that the influence of Nrf2 on the autophagy pathway has not been fully comprehended. Antioxidant defence and autophagy are both involved in maintaining homeostasis and cell survival but may interact differently depending on the level of stress, suggesting that Nrf2 may be an important sensor of cell survival/cell death^36^. The results of the MTT and Trypan blue assays showed that the DEP caused a significant decrease in cell viability, indicating their toxicity. Several studies have pointed out that the cytotoxicity induced by exposure to DEP can lead to cell death events such as apoptosis^37^ and necrosis^38^. Li *et al*.^39^ compared BEAS-2B cells and macrophages after exposure to DEP and demonstrated that BEAS-2B cells were more susceptible to the effect of the pollutant, causing a significant increase in necrotic cells. Seriani *et al*.^40^ demonstrated that, at the transcriptional level, BEAS-2B cells exposed to DEP at 15 μg/mL had significantly increased expression of the apoptotic protein caspase-3 and decreased expression of antioxidant enzymes, such as superoxide dismutase 1 and 2 (SOD1 and SOD2). Autophagy can be related as a cell death process, but is a different mechanism from apoptosis, also known as programmed cell death, characterized by several characteristic morphological changes in cell structure and with various enzyme-dependent biochemical processes^41^. However, in this study, cells had no apoptosis markers (data not shown) and had more than 70% of survival rate after DEP50 treatment and more than 90% after DEP10 treatment (Fig. 1), indicating that cells were on the threshold of a response to damage but were not on the threshold of death.

Therefore, the positive or negative regulation of autophagy and/or the antioxidant pathway can be attributed to the nature of the stimulus — and in the case of this study, to DEP, in particular. To determine whether the composition of the collected material was similar to that expected, the PAHs and elements were characterized (Tables 3 and 4). Although the characterization of DEP is the subject of studies seeking to understand how exposure causes adverse health effects^42^, no patterns of quality or quantity have been determined for the regulation of these specific elements in the air, making it difficult to determine the threshold of the amount of the element in cellular damage. Nevertheless, this is the first study to show a dependent relationship between autophagy and Nrf2 pathways after DEP exposure, indicating that the Nrf2 oxidative stress response positively regulates autophagy at the transcriptional level.

**Table 3:** Study Groups for BEAS-2B transfected.

Groups	Description
siRNA	BEAS-2B transfected with siRNA to Nrf2
siDEP10 1 h	BEAS-2B transfected and exposed to DEP10 µg/mL for 1 h
siDEP50 1 h	BEAS-2B transfected and exposed to DEP50 µg/mL for 1 h
siDEP10 2 h	BEAS-2B transfected and exposed to DEP10 µg/mL for 2 hs
siDEP50 2h	BEAS-2B transfected and exposed to DEP50 µg/mL for 2 hs
NTC – Negative Control	BEAS-2B transfected with negative control
DEP10 1 h	NTC BEAS-2B exposed to DEP 10 µg/mL for 1 h
DEP50 1 h	NTC BEAS-2B exposed to DEP 50 µg/mL for 1 h
DEP10 2 h	NTC BEAS-2B exposed to DEP 10 µg/mL for 2 hs
DEP50 2h	NTC BEAS-2B exposed to DEP 50 µg/mL for 2 hs

**Table 4:** Primers sequences.

Gene	Primer Forward	Primer Reverse
Nrf2	GCTATGGAGACACACTACTTGG	CCAGGACTTACAGGCAATTCT
Keap1	CGTCCTGCACAACTGTATCT	GTGTCTGTATCTGGGTCGTAAC
NQO1	GGAAGAAACGCCTGGAGAATA	CAGGGAAGCCTGGAAAGATAC
HO-1	GGTCCTTACACTCAGCTTTCT	CATAGGCTCCTTCCTCCTTTC
p62	GGAACAGATGGAGTCGGATAAC	CTGGAAGAAGGCAGAGAAACT
LCB3	AGAGCAGCATCCAACCAAAAT	TGAGCTGTAAGCGCCTTCTAA
ATG5	GCAACTCTGGATGGGATTGC	AGGTCTTTCAGTCGTTGTCTGAT
RPL13A	AAGGTGGTGGTCGTACGCTGTG	CGGGAAGGGTTGGTGTTCATCC

**Figure 6:**
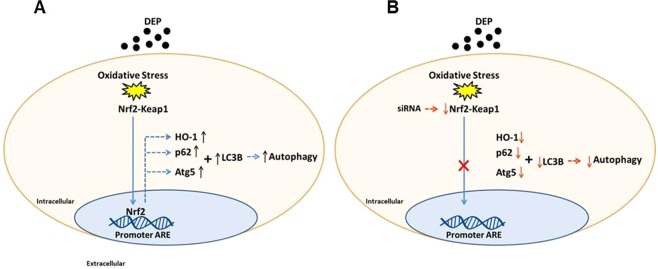
Summary of study completion. (**A**) DEP causes oxidative stress in cells, activating the Nrf2-system, which promotes antioxidant enzymes expression and positively regulates autophagy response. (**B**) under a decrease in Nrf2 expression, there is a drastic decrease in expression of antioxidant enzymes and autophagy markers.

In conclusion, this study showed that both the antioxidant defence promoted by Nrf2 and the autophagy mechanism acted together in the BEAS-2B cells exposed to diesel exhaust particulate matter through an increase in the expression of Nrf2, HO-1 and NQO1 and in the autophagy pathway proteins p62 and LC3B. However, the data available from the literature about how these mechanisms are related are conflicting. Our results indicated that the type of substance utilized (DEP) and the level of stress induced in the cell line used were determinants of the positive relationship between these pathways (Fig. 6). To test this hypothesis, further experiments are needed to determine whether any fraction or any specific component of the DEP is critical for the positive relationship between Nrf2 and autophagy presented here.

## Materials and Methods

This research was approved by the Ethical Committee of the School of Medicine of São Paulo University (number 119/15).

### Cell culture

A culture of BEAS-2B human bronchial epithelial cells, from an immortalized line (purchased from Sigma–Aldrich, Steinheim Germany, derived from normal bronchial epithelium obtained from autopsy of a non-cancerous individual) was prepared in flasks or plates coated with Matrigel (a substance that mimics the extracellular matrix) in the presence of LHC-9 growth medium supplemented with 10% foetal bovine serum, 0.1% 2 mM L-glutamine, 1% antibiotic, 0.1% 1 mM pyruvate, and 0.1% non-essential 1 mM amino acids. The bottles and plates were kept in a humidified CO_2_ incubator at 37 °C.

### Chemicals and treatment

Cells were exposed to bafilomycin (Sigma Aldrich) at 10 nM for 4 h as a negative control of autophagic pathways^43^. Exposure to EBSS (Earle’s balanced salt solution, Gibco) for 40 min was used to promote cell starvation. Sulforaphane (Sigma Aldrich) was used at 10 µM for 24 h as a positive control of the Nrf2 system^44^. All supplements were removed from the LHC-9 medium 2 h before each experiment.

### Diesel exhaust particulate and treatment

The DEP used in this study were collected from a diesel engine using a particulate retainer, a bimetallic filter that creates a field capable of holding the particulate matter emitted from exhaust. The vehicle chosen was a 2004 model Volkswagen bus with a 6-cylinder MWM x-10 engine. The bus is the property of a private company and circulates in the neighbourhood of Campo Belo, in Sao Paulo City, Brazil. The bus runs approximately 100 to 150 km/day, and the particle retainer was linked to the exhaust for 1 week. DEP were collected in September/October of 2015. Next, the DEP were removed from the particle retainer and weighed. For BEAS-2B cell exposure, the DEP were diluted 1:1 (1 mg/mL) in phosphate-saline solution (PBS) with 0.1% ethanol. The DEP were diluted in culture medium at concentrations of 10, 50, and 100 μg/mL.

### Diesel exhaust characterization

As recommended by Laks *et al*.^45^, the elements that compose the material were determined using X-ray fluorescence and the energy dispersive method (EDX). As previously described^46^, the HPA fractions and their derivatives were separated by high-performance liquid chromatography, and for the identification and quantification of these HPA fractions, gas chromatography was used in performed in conjunction ion with mass spectrometry (GC/MS).

### Cytotoxicity assays

#### MTT [3-(4,5-dimethylthiazol-2-yl)-2,5-diphenyltetrazolium bromide] assay

In a 96-well plate, 3 × 10^4^ cells were seeded in each well, and DEP suspensions were added at 10, 50, and 100 μg/mL concentrations. The cells were exposed at time intervals of 20 min, 40 min, 1 h and 2 h. After the cells were exposure to DEP, the wells were washed with PBS. A solution with 2.5 mg/mL MTT (20%) was added into each well, and the cells were incubated for 4 h in a CO_2_ incubator. After 4 h, the cells in the plate were homogenized with DMSO for 30 min. The plates were read using an enzyme-linked immunosorbent assay (ELISA) reader at 570 and 630 nm. The data for each concentration and time were collected in triplicate and relative O.D were calculated to the control.

#### Trypan blue assay

In a 12-well plate, 3 × 10^4^ cells were seeded in each well with LHC-9 medium. The cells were exposed to DEP at 50 and 100 μg/mL concentrations for 1 and 2 h. At the end of the experiment, the cells were washed with 1 × PBS and subjected to trypsin. Next, the cells were collected in a sterile tube and centrifuged for 3 min at 5000 rpm. The supernatant was discarded, and the cells were resuspended in a Trypan Blue dye solution. Ten microliters of this dyed cell mixture was placed on a Countess slide (Invitrogen). The data for each concentration and time were collected in triplicate.

### BEAS-2B cell transfection

To further investigate the effect of Nrf2 on the autophagic pathway in BEAS-2B cells exposed to DEP, silencing by interference RNA (siRNA) was used to degrade the Nrf2 mRNA to prevent its overexpression and to verify the mechanism through which the autophagy pathway is regulated under these conditions. Lipofectamine RNAiMAX (Invitrogen), siRNA sequences s9394 (Ambion Silencer Select siRNA) and s9491 (Ambion Silencer Select siRNA) and a negative control (Ambion Silencer Select Negative Control) were each utilized at a concentration of 20 nM^44,47^. The negative control (NTC) simulates the same transfection conditions; however, the transfected RNA had a non-significant sequence. To facilitate transfection, siRNA and Lipofectamine were incubated with Opti-MEM medium for 5 min and added to the cell culture medium as instructed by the manufacturer and incubated for 48 h to complete the transfections. The groups of BEAS-2B cells are described in Table 3.

#### RT-PCR

BEAS-2B and siRNA-transfected BEAS-2B cells were seeded separately in 96-well plates at 3 × 10^4^ cells per well in triplicate for each cell group. After exposure to the treatments (DEP, sulforaphane, EBSS and bafilomycin), total RNA was extracted from the cell pellet using 700 μL of Qiazol reagent (Qiagen) with an extraction column (RNeasy Mini kit Qiagen), according to the manufacturer’s instructions. The RNA was stored at −80 °C. The complementary DNA (cDNA) was synthesized using 200 ng of total RNA incubated in the presence of 4 μl SuperScript VILO Master Mix (Invitrogen) according to the manufacturer’s instructions. All samples were processed for RT-PCR in quadruplicate in a Rotor Gene 3000 instrument (Qiagen). The analysis was performed by calculating the relative quantity of each gene of interest in relation to a housekeeping gene, as indicated in the model described by Livak and Schmittgen^48^ and using the 2^−ΔΔCT^ formula. The primer sequences are listed in Table 4. All 2^−ΔΔCT^ values are informed in Supplementary Material.

### Statistical analysis

The data distribution was verified by Kolmogorov-Smirnov analysis. The normally distributed variables were analysed by one-way ANOVA, and for the non-normally distributed variables, the non-parametric Kruskal-Wallis test was used. The data are presented in box plot graphs, with median and interquartile range (25% and 75%). “Pairwise” and “Holm-Sidak” multiple comparison tests were used to detect differences between groups. The analyses were performed with Sigma Plot and IBM SPSS 21.0 statistical software. The level of significance was considered to be p ≤ 0.05.

### Western blotting

BEAS-2B and siRNA-transfected BEAS-2B cells were seeded separately in 6-well plates, containing 2 × 10^5^ cells per well, in duplicate per group. Following cell exposure, the proteins were collected with RIPA extraction buffer in the presence of protease inhibitors and phosphatase inhibitors (Sigma Aldrich). Total protein was quantified by the Bradford method, and 50 μg of protein was used for electrophoresis on a 12% polyacrylamide gel. After the proteins were transferred to a PVDF membrane, the membrane was blocked in non-fat dry milk diluted to 5% in PBS with 0.1% Tween for 1 h. After blocking, the membrane was incubated overnight with antibodies against β-actin (Sigma 1:10000), Nrf2 (Abcam 1:1000), LC3B (Abcam 1:1000) and p62 (Abcam 1:1000). Next, the membrane was washed 3 times with PBS-Tween and incubated with goat anti-rabbit HRP secondary antibody (1:1000) for 1 h. The membrane was then revealed by chemiluminescence using an ECL kit (GE Healthcare Life Sciences) on an ImageQuant™ LAS 4000 apparatus. Every image was captured after 10–30 seconds of exposure. The proportion of each protein was obtained by the densitometry method. ImageJ software was used to analyse the intensity of the bands. On the basis of the band area values, the proteins of interest were normalized to β-actin expression (area of interest protein/β-actin area). After normalization, the ratio (R) of each treatment was calculated according to the value of the untreated cell (treatment/untreated, in which untreated cell = 1). Thus, an increase in the protein is considered when R > 1. Each analysis was performed in duplicate.

### siRNA immunofluorescence assay

BEAS-2B and siRNA-transfected BEAS-2B cells were seeded separately in 24-well plates at 2 × 10^5^ per well in duplicate per group with coverslips in the wells for use in microscopy. After exposure, the cells were fixed with 100% methanol for 15 min at −20 °C and then washed 3 times with PBS for 10 min at 4 °C. For permeabilization, the samples were incubated for 10 min with PBS with 0.2% Triton X-100. Next, the samples were blocked for 1 h with 5% BSA and incubated overnight at 4 °C in a humid chamber with primary antibodies against LC3B (Cell Signaling 1:100) and Nrf2 (Abcam 1:50) for double staining. On the following day, the samples were incubated with secondary antibodies (Alexa Fluor-488 and Alexa Fluor-546, 1:200) for 1 h in a humid chamber at room temperature and then incubated with 1:200 DAPI for 15 min. The images were captured under a 40× objective lens with Image Pro Plus software. After being acquired, the photos were merged by ImageJ software to combine the green, red and blue markers. The chosen fields were augmented 2×. Antibodies control test is available in Supplementary Material.

## Supplementary information

Supplementary Material.
