# Supplementary material for: Nrf2 positively regulates autophagy antioxidant response in human bronchial epithelial cells exposed to diesel exhaust particles
Source: Sci Rep. 2020 Feb 28;10:3704. doi: 10.1038/s41598-020-59930-3 (PMC7048799; doi:10.1038/s41598-020-59930-3)

Nrf2 positively regulates autophagy antioxidant response in human bronchial epithelial cells exposed to diesel exhaust particles.

Daniela Perroni Frias[a*]; Raquel Labiapari Nunes [a]; Kelly Yoshizaki[a]; Regiani Carvalho-Oliveira[a]; Monique Matsuda[b]; Mara de Souza Junqueira[c]; Walcy Rosolia Teodoro[d]; Pérola de Castro Vasconcellos[e]; Daniela Cristina de Almeida Pereira[e]; Paulo Roberto da Conceição[f]; Paulo Hilário do Nascimento Saldiva[a]; Thais Mauad[a]; Mariangela Macchione[a].

Journal: Scientific Reports

*Corresponding author:

Contact: Daniela Perroni Frias, MSc

Tel: 55 11 30618520

e-mail: danielapfrias@usp.br

Supplementary Data

Tables 5 to 9: show the significant values of p presented by the multiple comparison test Pairwise.

Table 10: shows the sample/control ratios of the bands density of Nrf2, p62, LC3I and LC3II proteins normalized by β-actin.

Table 11 to 13: shows values to Relative Concentration of RNAm by $2^{-\Delta\Delta CT}$ method.

Table 14: shows normalized (using b-actin) densitometry values for the two Western Blotting replicates (2018 and 2019).

Figure 1: Hep2 slides were used as positive controls to LC3B and Nrf2 antibody, as suggested by providers.

Table 5 – Significant values of p for expression of Nrf2

Nrf2 Expression	
**Paired Groups**	**p value**

siRNA vs NTC	0.027
siRNA vs DEP50 1h	0.011
siRNA vs DEP10 2h	0.001
siRNA vs DEP50 2h	0.001
siDEP10 1h vs NTC	0.007
siDEP10 1h vs DEP10 1h	0.018
siDEP10 1h vs DEP50 1h	0.002
siDEP10 1h vs DEP10 2h	<0.001
siDEP10 1h vs DEP50 2h	<0.001
siDEP50 1h vs NTC	0.018
siDEP50 1h vs DEP10 1h	0.043
siDEP50 1h vs DEP50 1h	0.007
siDEP50 1h vs DEP10 2h	<0.001
siDEP50 1h vs DEP50 2h	0.001
siDEP10 2h vs DEP10 2h	0.022
siDEP10 2h vs DEP50 2h	0.023
siDEP50 2h vs DEP10 2h	0.01
siDEP50 2h vs DEP50 2h	0.011

Table 6 – Significant values of p for expression of HO-1

HO-1 Expression	
**Paired Groups**	**p value**
siRNA vs DEP10 2h	0.026
siRNA vs DEP50 2h	0.007
siDEP10 1h vs NTC	0.008
siDEP10 1h vs DEP10 1h	0.001
siDEP10 1h vs DEP50 1h	0.005
siDEP10 1h vs DEP10 2h	<0.001
siDEP10 1h vs DEP50 2h	<0.001
siDEP50 1h vs NTC	0.031
siDEP50 1h vs DEP10 1h	0.006
siDEP50 1h vs DEP50 1h	0.021
siDEP50 1h vs DEP10 2h	0.002
siDEP50 1h vs DEP50 2h	<0.001
siDEP10 2h vs DEP50 2h	0.016
siDEP50 2h vs DEP10 1h	0.013
siDEP50 2h vs DEP50 1h	0.038
siDEP50 2h vs DEP10 2h	0.05
siDEP50 2h vs DEP50 2h	0.001

Table 7 – Significant values of p for expression of LC3B

LC3B Expression	
**Paired Groups**	**p value**
siRNA vs DEP50 1h	0.005
siRNA vs DEP10 2h	0.034
siDEP10 1h vs NTC	0.013
siDEP10 1h vs DEP10 1h	0.031
siDEP10 1h vs DEP50 1h	<0.001
siDEP10 1h vs DEP10 2h	0.004
siDEP10 1h vs DEP50 2h	0.008
siDEP50 1h vs NTC	0.024
siDEP50 1h vs DEP50 1h	0.001
siDEP50 1h vs DEP10 2h	0.008
siDEP50 1h vs DEP50 2h	0.016
siDEP10 2h vs NTC	0.015
siDEP10 2h vs DEP10 1h	0.036
siDEP10 2h vs DEP50 1h	<0.001
siDEP10 2h vs DEP10 2h	0.005
siDEP10 2h vs DEP50 2h	0.009
siDEP50 2h vs DEP50 1h	0.003
siDEP50 2h vs DEP10 2h	0.023
siDEP50 2h vs DEP50 2h	0.04

Table 8 – Significant values of p for expression of p62

p62 Expression	
**Paired Groups**	**p value**
siRNA vs DEP50 1h	0.01
siRNA vs DEP10 2h	0.001
siRNA vs DEP50 2h	0.001
siDEP10 1h vs DEP50 1h	0.006
siDEP10 1h vs DEP10 2h	0.001
siDEP10 1h vs DEP50 2h	0.001
siDEP50 1h vs DEP50 1h	0.025
siDEP50 1h vs DEP10 2h	0.004
siDEP50 1h vs DEP50 2h	0.004
siDEP10 2h vs DEP10 2h	0.016

siDEP10 2h vs DEP50 2h	0.017
siDEP50 2h vs DEP10 2h	0.013
siDEP50 2h vs DEP50 2h	0.014
DEP10 1h vs DEP10 2h	0.029
DEP10 1h vs DEP50 2h	0.032

Tabela 9 – Significant values of p for expression of Ag5

Atg5 Expression	
**Paired Groups**	**p value**
siDEP10 1h vs NTC	0.007
siDEP10 1h vs DEP50 1h	0.046
siDEP10 1h vs DEP10 2h	<0.001
siDEP10 1h vs DEP50 2h	0.019
siDEP50 1h vs DEP10 2h	0.009
siDEP10 2h vs NTC	0.037
siDEP10 2h vs DEP10 2h	0.004
siDEP50 2h vs NTC	0.002
siDEP50 2h vs DEP50 1h	0.014
siDEP50 2h vs DEP10 2h	<0.001
siDEP50 2h vs DEP50 2h	0.005
DEP10 1h vs DEP10 2h	0.007
siDEP50 2h vs siRNA	0.024

Table 10: R Values

	**Nrf2**	**p62**	**LC3I**	**LC3II**
NTC	1.000	1.000	1.000	1.000
DEP10 1h	1.096	1.333	1.463	1.463
DEP50 1h	0.695	0.824	1.092	1.329
DEP10 2h	0.445	2.092	1.430	1.180
DEP50 2h	1.425	0.431	0.970	0.956
siRNA	0.877	0.415	0.187	0.774
siDEP10 1h	0.474	0.890	0.235	0.556
siDEP50 1h	0.369	0.619	0.257	0.775
siDEP10 2h	0.411	0.567	0.175	0.374
siDEP50 2h	0.259	0.259	0.075	0.218

Table 11: $2^{-\Delta\Delta CT}$ values for control, bafilomycin, EBSS and sulforaphane treatments

	Nrf2	NQO1	HO-1	p62	LC3B	Atg5
Control	0.83	0.9	0.98	0.94	0.94	1.22
	0.99	1.04	0.85	0.79	0.8	1.00
	1.22	1.16	0.91	1.2	1.06	0.67
	0.89	0.94	1.03	1.19	1.01	1.44
	1.01	0.8	1.06	1.11	0.94	1.07
Baf10	0.61	0.47	0.3	0.77	0.45	0.64
	0.68	0.55	0.35	0.86	0.52	1.01
	0.7	0.62	0.31	0.82	0.58	0.97
	0.66	0.6	0.3	0.67	0.62	1.32
EBSS	0.82	0.94	1.06	0.49	0.82	1.22
	1.09	1.17	1.31	0.61	1.09	1.46
	1.09	1.18	1.37	0.57	1.09	1.39
	0.97	1.14	1.19	0.55	0.97	1.4
Sulf10	1.00	2.22	2.18	0.83	1.42	1.72
	1.19	2.17	2.49	0.99	1.66	1.74
	1.07	2.22	2.6	1.22	1.69	2.53
	1.02	2.07	2.44	1.49	1.41	3.14

Table 12: $2^{-\Delta\Delta CT}$ values to DEP treatment for 2 hours

	LC3B	p62	Nrf2	NQO1	Atg5	HO-1
Control	0.93	0.79	0.82	0.67	1.09	1.36
	1.05	1.14	1.12	0.74	0.95	1.42
	1.31	1.14	1.15	1.14	0.97	1.24
	0.78	0.98	0.95	0.87	0.59	0.99
10µg/mL 2h	1.00	2.71	1.91	0.95	0.77	3.01
	1.44	2.44	1.76	0.9	0.79	2.69
	1.08	2.15	2.04	0.81	0.74	2.71
	1.17	2.02	2.12	0.65	0.81	1.74
50µg/mL 2h	2.1	3.4	2.24	1.71	0.77	3.48
	2.29	3.99	3.4	1.96	1.28	4.96
	3.69	4.34	4.1	2.1	1.53	6.02
	3.56	4.07	3.57	2.22	1.95	6.28

Table 13: $2^{-\Delta\Delta CT}$ values to NTC and siRNA groups

	LC3B	p62	Atg5	Nrf2	HO-1
NTC	1.5	1.31	0.93	1.4	1.00
	1.48	1.44	1.06	1.48	1.01
	1.56	1.65	1.09	1.57	0.93
	1.91	2.29	1.79	2.17	1.18
DEP10 1h	1.59	1.78	0.8	1.39	1.25
	1.43	1.69	0.66	1.44	1.02
	1.32	1.57	0.73	1.65	1.14
	1.67	0.61	0.73	1.49	1.16
DEP50 1h	1.91	2.62	1.12	1.95	1.16
	1.82	2.22	1.11	1.96	1.07
	1.81	2.17	0.79	1.72	1.04
	1.98	2.14	0.84	1.86	0.84
DEP10 2h	1.51	2.33	1.67	2.3	1.01
	1.75	2.38	1.66	2.01	1.12
	1.88	3.15	1.77	2.81	1.61
	1.63	4.04	2.17	2.41	1.58
DEP50 2h	1.81	2.63	0.87	2.11	1.59
	1.7	2.18	0.85	2.1	1.35
	1.59	3.17	1.23	2.71	1.32
	1.49	3.98	1.29	2.53	1.54
siRNA	0.67	0.91	0.85	0.2	0.43
	0.68	1.01	0.99	0.2	0.47
	0.92	1.07	0.91	0.21	0.33
	1.03	1.11	0.86	0.18	0.33
siDEP10 1h	0.66	1.1	0.54	0.16	0.2
	0.66	1.23	0.5	0.16	0.21
	0.69	0.76	0.55	0.17	0.19
	0.63	0.87	0.62	0.16	0.22
siDEP50 1h	1.12	0.88	0.39	0.14	0.22
	0.69	0.82	0.38	0.13	0.18
	0.61	1.22	0.93	0.22	0.3
	0.62	1.83	1.08	0.3	0.4
siDEP10 2h	0.77	0.9	0.47	0.3	0.37
	0.8	1.1	0.61	0.28	0.4

siDEP50 2h	0.59	1.47	0.69	0.39	0.59
	0.51	2.02	0.93	0.44	0.69
	0.89	1.3	0.34	0.26	0.38
	0.79	1.11	0.36	0.26	0.26
	0.76	1.38	0.44	0.32	0.29
	0.65	1.1	0.63	0.33	0.3

Table 14: densitometry values for Western Blotting replicates

		Nrf2	p62	LC3I	LC3II
Replicate 2018	NTC	0.91	1.48	1.11	1.06
	DEP10 1h	0.50	0.50	1.03	1.52
	DEP50 1h	0.31	1.24	0.86	1.14
	DEP10 2h	0.37	0.92	1.04	1.09
	DEP50 2h	0.41	1.41	0.92	0.99
	siRNA	0.51	0.99	0.19	0.22
	siDEP10 1h	0.94	0.88	0.10	0.66
	siDEP50 1h	0.49	0.63	0.60	0.43
	siDEP10 2h	0.32	0.63	0.44	0.49
	siDEP50 2h	0.16	0.13	0.55	0.76
Replicate 2019	NTC	1.50	1.43	0.91	1.43
	DEP10 1h	1.64	2.10	1.21	2.09
	DEP50 1h	1.04	1.90	0.75	1.56
	DEP10 2h	0.67	1.69	1.90	2.04
	DEP50 2h	2.14	1.37	0.39	1.38
	siRNA	1.32	1.11	0.38	0.27
	siDEP10 1h	0.71	0.80	0.81	0.33
	siDEP50 1h	0.55	1.11	0.56	0.37
	siDEP10 2h	0.62	0.54	0.52	0.25
	siDEP50 2h	0.39	0.31	0.24	0.11

Figure 1: Nrf2 and LC3B antibody tests

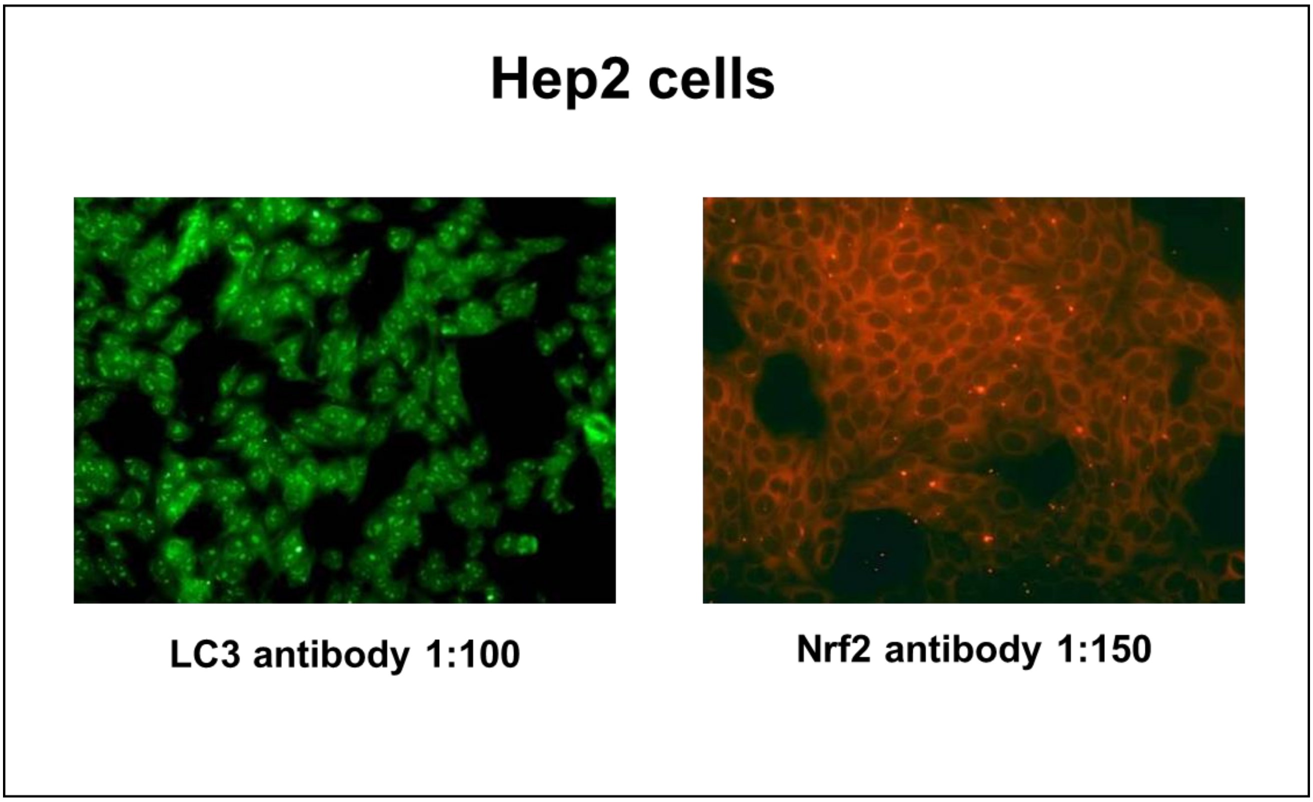

Supplement: Supplementary file 1 — Supplementary Material. [file 41598_2020_59930_MOESM1_ESM.pdf]
